# Address

**Published:** 1883-08

**Authors:** J. Hooper


### Address.

BY J. HOOPER.

[Prest. Ky. State Dental Society.]

Gentlemen:—In looking over this audience to-day, a feeling of sadness wells up in my heart in the contemplation of that one who must ever be absent from our association. There is in our midst a vacant chair, never to be filled. A voice which once united with us in council, and ever pleasantly greated us with, "How are you, my brother," is silent. A smile of kindly welcome hath departed, and these but remind us of the sad loss of our dear friend, William H. Goddard, or "Pap Goddard," as we usually greeted him, and truly; for was he not a father to the younger men of the dental profession? He entered it before it was a science—rather, when a mechanical trade, and left an example the younger members should endeavor to follow by ever being upon the alert when opportunity proffered advancement to the science of dentistry. He lent a willing hand and heart to the chariot wheel of dental science whenever required, to raise it from the mire of charlatanism. He fell while standing on the very highest pinnacle upon which the profession could place him, holding high our noble banner, and bequeathing to us an example worthy of our emulation. While we deeply feel our loss and must ever revere his memory, we must not repine at the workings of a higher power, but ever plead in the cause of the progress of dentistry, which always gained his approval. To mother earth we consigned his loved form and to his Maker his spirit. Our legacy from him, is onward and upward.

In reviewing the various fields of dental science, I note some that should be more thoroughly cultivated, to which I desire to call the attention of this association, that the mind of the public may be aroused and educated to the extent that it may be enabled to discriminate between scientific and false dentistry, and be protected from imposition and fraud, thereby advancing and elevating the science.

Could the public mind be trained thus, there would be a demand for well qualified dentists, and stimulate those who are indifferent to higher aims in the profession.

But to accomplish this, will require the hearty co-operation of all desirous of attaining for our cause the highest position, and to do this the "ax and hoe" in this field must be wielded by strong hands, and backed by willing hearts.

Scientific facts must be established that cannot be refuted. We want men of courage, strength of character and will, to lead us, who bear upon their banner the motto, "I will succeed;" not as some have done, labor industriously for awhile, then turn aside to some other field because of its popularity. In this connection, I will mention those of the greater import.

First—Food and its relation to the teeth.

Second—Eruption of the teeth.

Third—Care of school children's teeth.

Fourth—Educating the public.

How important to cultivate scientifically the first field mentioned. The whole animal and vegetable kingdoms owe their life and existence to food, being composed of elements obtained from it; and when I say food, I mean that which we eat, drink, breathe and absorb for the sustenance of our bodies.

Dr. E. S. Gaillard says, in an article on food, that it can be divided into two classes—the nitrogenized and non-nitrogenized, the former class being that which contains gluten, forming the teeth, bone and muscle, and imparting to them their strength. The latter class starch, sugars, and all classes of sweets which form the fats for the purpose of maintaining animal temperature.

It is not surprising to hear the question from our patients, "Why are my teeth so imperfect, when my parents and grandparents had good teeth?"--when we consider that there are ten pounds of non-nitrogenized food eaten now where there was one in our forefathers' day. The food at that time was not deprived of its gluten, or nitrogen, as at the present day.

I will append a list of some of the cereals that have been analyzed by Dr. Ephriam Cutter, A.M., M.D., of New York (late of Boston), and published by Dr. E. S. Gaillard, A.M., M.D., LL.D., of New York. He says: "The following advertised food stuffs contain no gluten cells: Cold blast flour, extra; barley flour, extra; buckwheat flour, Indian wheat flour, Lost Nation wheat flour, Minnesota Surprise flour, Hazleton wheat flour, Puritan wheat flour, Patapsco flour, Underwood wheat flour, fine granulated wheat flour, Gerber's food for infants and children, and many other preparations, contain no gluten cells." He further states: "The medical profession should establish an accurate public sentiment as regards cereal foods; those lacking the gluten cells are devoid of the nutritive elements of the grain." All scientific professions should enlighten the public mind on this all-important subject.

Dr. Gaillard cites a well-known physician of New York, who endeavored to ascertain the duration of time he could maintain life, without serious suffering, on a diet of gum and starch; though the idea was soon abandoned, he has never recovered from the effects of his rash experiment.

Twenty-five years have elapsed since the endurance of his self-imposed ordeal, and to-day his heart beats thirty per cent. faster than it did previous to the experiment.

First—Dentition is the most critical period of the human family, the mortality being greater at that date than any other, attributed to dentition. Medical and dental professions should especially cultivate this field; it belongs rather to the former in public opinion, because it has been misplaced, but should be recognized as properly belonging to the dental profession. I believe if dentists had charge of first dentition the mortality of children would be decreased. The study of the teeth makes dentistry a profession. Some physicians will tell you that the child's gums should not be lanced to avoid the formation of a cicatrix, but when a man who has been a hard student and close observer for over thirty years, and is accepted as authority, informs us we need not fear that; that he has never found a cicatrix in all those years, I think he may safely be relied upon.

A few words on the care of children's teeth between the ages of three and five years. A great many lose their teeth during this period. If parents knew what could be done, and the importance of caring for them, I know they would willingly do it. If extracted before the proper time there is great danger of rupturing the gums of the permanent teeth, interfering with mastication, and causing contraction of the arch, producing deformity.

The case of school children's teeth is attracting more universal attention. We begin with children at the age of five and continue until eighteen or twenty, during which children in this country attend school. At the age of six they begin to erupt the permanent teeth, and during that period nature is very much taxed for the general development of the entire body, and it is vastly important that due regard be given the teeth at this stage of existence, that they may masticate the food properly, that it may assimilate and build up the body as nature demands.

If neglected, which 90 per cent. are, the result will be disastrous, which I will prove by competent witnesses before I finish this subject. I place the per cent. of decayed, or carious, teeth somewhere in seventy; those affected by tartar in fifty; diseased gums, between thirty and forty, and irregularities between twenty and thirty.

All dentists, who have been close students and observers, as well as parents, who have their children's teeth examined, will bear me witness and testify to the disastrous results occurring from the neglect of teeth.

The eyes and teeth are supplied by the same nerve, or a branch of the nerve, and a scientific oculist will inform you that diseased teeth frequently produce serious trouble of the eyes. I could refer to many, both occulists and dentists, as authority for the truth of this assertion. I have frequently had children sent to me (to treat or extract their teeth) before the oculist could treat them for diseased eyes.

There is much discomfort produced from an unhealthy mouth. A child is unfit for school duties who is so unfortunate as to suffer from this cause, and such a condition renders him dull and stupid. He cannot masticate his food, which provokes indigestion, entailing upon him many ills to which human flesh is subject. An adult in such a state is unfit for business and society. It is useless to endeavor to force knowledge into brains unfit to receive it, and using school funds in that manner is actual robbery. One fourth of the means employed to establish dental boards or employ dentists to examine or keep in a healthy state the children's teeth would be a saving of money and gaining of time. Children would learn faster from being in a condition to digest what they study, and the entire country would be benefitted. Gentlemen, by the united effort we can establish such a course. We must do it. We owe it to dentistry and the public. I desire this association to appoint a committee to take charge of this matter, gain all the knowledge they can on the subject, have the facts published in pamphlet form, and give the public the benefit of it. Each School Trustee in the State should possess a copy, and every member of the Legislature, and that committee should visit that body and endeavor to enlist their assistance in this grand cause.

The time is approaching when we must have legislation on this point. Why delay it?

Why should not the grand old State of Kentucky stand in the foremost rank? In France the authorities have taken steps in this direction. They have an established system by which the school children's teeth are examined periodically, and great good must spring from such a course. Why then, I ask, must we be tardy in action? Dr. Gross, of Philadelphia, some two years ago recognized the great importance of our profession when he addressed the American Medical Association, which at that time convened at Richmond, Va., and was the cause of the formation of the "Section on Dentistry." He said truly: "Every man, woman and child in the civilized world requires the services of the dentist, whereas comparatively few persons require the services of the oculist, the aurist, or the laryngologist."

Fourth—How to educate the public. The press stands first as the great educator in this enlightened and progressive age. If it should become interested and united upon any subject in any State or county, what a power might it not wield for good! Executive officers of cities, Congress, of our States, and even the President, the highest officer of our land, bow at its shrine. Gentlemen, you see and feel its influence.

The press once enlisted in our cause to send these facts broadcast over our glorious land, the public would demand that members of the profession would uphold it by being active in its advancement, knowing that justice would then be given at the hand of the dentist.

The last improvement meets approval. Who prefers not the modern travel to that of former times. Away with the coach, on with the locomotive. There is as great difference between ancient and modern dentistry as ancient and modern travel. It has been asserted that dentistry has made greater progress than any of the known sciences.

The time is approaching when every medical school will have a dental professorship. Why can not Louisville establish one and be foremost in the ranks?

It has been said, and truly, that "the mouth is the index of a great many diseases." The practitioner of medicine should then be perfectly familiar with the index of those diseases.

---

Sounds from the Consulting Room.—"How long will it take you to cure me, doctor?" "Well, Mr. Blank, I think you can get back to your desk at the bank in about a month, but you will have to remain on treatment for several years." "But, you mistake; I am not Mr. Blank the banker, but Mr. Blank the letter-carrier." "Oh! that alters the case. There is nothing the matter with you but a little biliousness. You will be all right in a month.

---

Among the million of paupers aided by the public charities of England in 1865 there were eight hundred thousand drunkards.

